# The reintroduction of hemp in the USA: a content analysis of state and tribal hemp production plans

**DOI:** 10.1186/s42238-023-00181-0

**Published:** 2023-06-07

**Authors:** Amanda Falkner, Jane Kolodinsky, Tyler Mark, William Snell, Rebecca Hill, Amelia Luke, Jonathan Shepherd, Hannah Lacasse

**Affiliations:** 1 Department of Community Development and Applied Economics, University of Vermont, 146 University Place, Morrill Hall Room 205, Burlington, VT 05405-1016 USA; 2 University of Kentucky, Lexington, USA; 3 Colorado State University, Fort Collins, USA

**Keywords:** Hemp, Cannabis, United States Department of Agriculture, Farm Bill, Content analysis, Regulation, Cannabis plant

## Abstract

**Background:**

The reintroduction of *Cannabis sativa L*. in the form of hemp (< 0.3% THC by dry weight) into the US agricultural sector has been complex and remains confounded by its association with cannabis (> 0.3% THC by dry weight). This has been further exacerbated by inconsistent hemp regulations in the US since the 2014 Farm Bill’s reintroduction.

**Methods:**

A content analysis was performed to analyze the terms and definitions presented by state and tribal hemp production plans, the USDA Hemp producer license, and the 2014 state pilot plans. A total of 69 hemp production plans were analyzed.

**Results:**

Results suggest significant discrepancies between hemp production plans, which have been exacerbated by extending the 2014 Farm Bill language into the 2018 Farm Bill timeframe.

**Conclusions:**

Findings from this study point to areas in need of uniformity and consistency as the regulatory framework is modified and provides a starting point for change for federal policymakers. The results may also be useful to companies attempting to market products across state boundaries. Suggestions for how to mitigate these inconsistencies are provided based on the content analysis findings.

**Supplementary Information:**

The online version contains supplementary material available at 10.1186/s42238-023-00181-0.

## Background

In 1937, the Marihuana Tax Act imposed taxes on the sale of *Cannabis sativa L*. (cannabis), discouraging its production due to the failure of the Act to differentiate between hemp and cannabis (United States Department of Agriculture Economic Research Service 2000). Botanically, both hemp and cannabis are derived from the same plant: *Cannabis sativa L*. Attitudes toward cannabis were further tainted when it was classified as a Schedule I drug in 1970 under the Controlled Substances Act, which effectively made its production illegal (Johnson 2018; Malone and Gomez 2019). This remained in place until 2014, with the passage of the Farm Bill, when a distinction between hemp and cannabis was established for US producers through the “Legitimacy of Hemp Research” pilot program (Pal and Lucia 2019).

The United States Department of Agriculture (USDA) formally distinguishes the two plants based on THC (Δ^9^-tetrahydrocannabinol) content, which is the primary psychoactive component of the *Cannabis sativa L*. plant (U.S Drug Enforcement Administration 2020). A plant is considered hemp if the THC content is less than 0.3% on a dry weight basis and is considered cannabis if the THC content exceeds the 0.3% threshold (Johnson 2019). Utilizing this definition, state-level legislation was passed to develop pilot programs for hemp production in 2014 (Agricultural Act 2014). The pilot program opened up the opportunity for farmers to grow hemp for the grain, floral, and fiber markets. Four years later, the 2018 Farm Bill removed hemp’s Schedule I drug classification, re-legalizing its production in the USA after a more than 45-year ban (Johnson 2019). However, the reintroduction of the crop into the agricultural sector has been complex and remains confounded by its association with cannabis (Campbell et al. 2021).

The reintroduction of hemp to the US agricultural landscape with the passing of the 2014 Farm Bill was a momentous development. While the pilot program offered each state the autonomy to create individualized hemp production plans, this proved to have its downfalls. While this allowed for individual adjustments to the hemp production plans to fit their needs, it created an opportunity for each state to maximize its competitiveness within the burgeoning industry, which may have actually impeded industry development and growth (Mark et al. 2020). For instance, states implemented different THC testing protocols, licensing fees, sampling procedures, and data collection. This resulted in a patchwork of hemp legislation across the country that was inconsistent in its terminology and processes, threatening the viability of this new sector and creating many challenges for both producers and hemp businesses in marketing products across state lines (Mark et al. 2020).

The 2018 Farm Bill made significant changes to the existing regulatory framework the 2014 pilot plan set forth, further complicating the existing disparities across state plans. First, it broadened the scope to include tribal governments, whereas previous regulations had only allowed states to develop independent plans (Agricultural Act 2018). Additionally, it created the interim final rule and final rule for hemp production, resulting in the USDA Hemp Producer License under which states and tribal governments could choose to operate. Enacted on March 22, 2021, the final rule for hemp production partially clarified the regulation requirements for US state and tribal governments by providing regulatory guidance from a Federal level (U.S. Department of Agriculture Agricultural Marketing Service 2021). Regulatory guidance provided by the final rule included direction on performance testing standards, sampling standards, harvest window, sampling agent requirements, and testing labs. To adhere to this guidance, state and tribal governments have been allowed to submit initial or revised individualized plans that incorporate the USDA Hemp Producer License regulations and include any clauses specific to their needs. While this allows for individual amendments, it has also perpetuated the ongoing lack of consistency between plans.

**Fig. 1:**
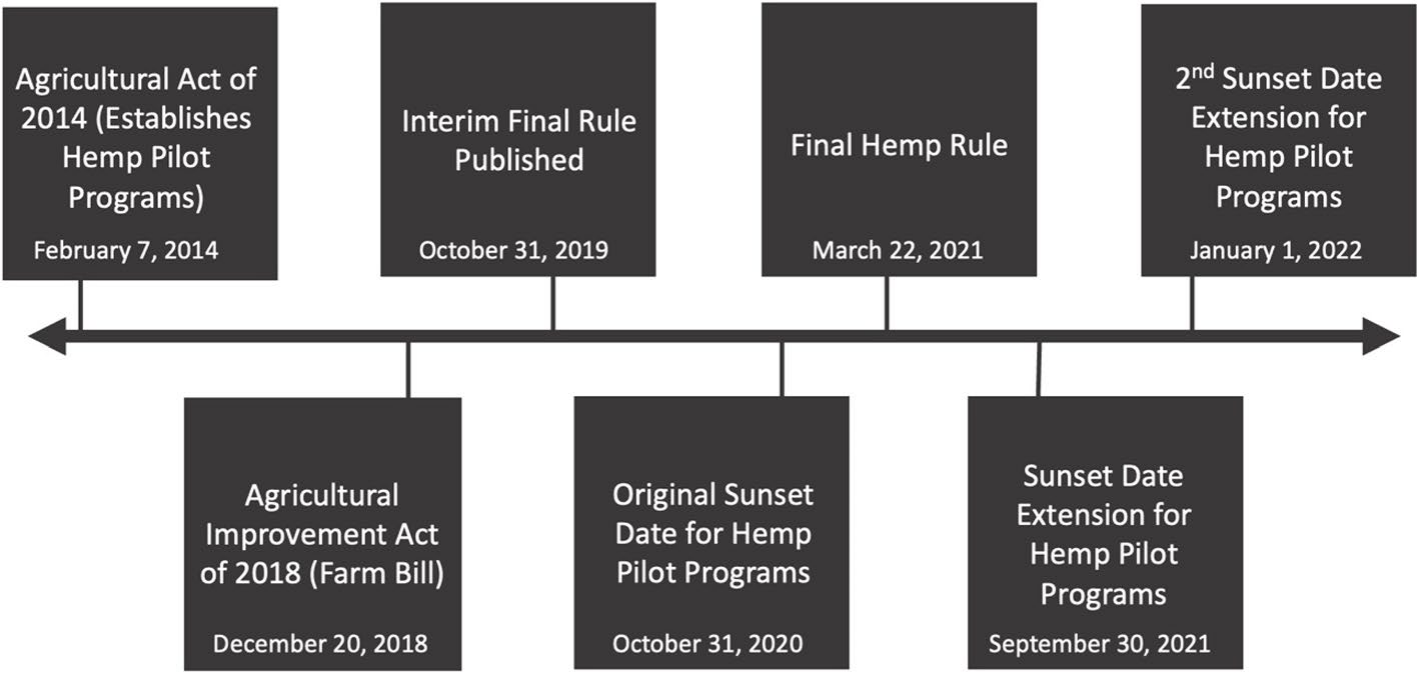
Hemp policy timeline. Adapted from: US Department of Agriculture Agricultural Marketing Service, 2021. This timeline includes important dates of policy changes and adaptations since the reintroduction of hemp production in the 2014 Farm Bill through pilot plans. The 2018 Farm Bill made significant changes to the existing regulatory framework of the 2014 pilot plan by broadening the scope to include tribal governments, whereas previous regulations had only allowed states to develop independent plans. The 2018 Farm Bill also created the interim final rule and final rule for hemp production, resulting in the USDA Hemp Producer License under which states and tribal governments could choose to operate. The final rule for hemp production partially clarified the regulation requirements for US state and tribal governments by providing a regulatory baseline that must be adhered to. The sunset date is the date when language from the 2014 Farm Bill pilot programs was set to expire and was no longer considered valid, requiring that producers either adopt the USDA hemp producer license or submit an independent production plan for review. The original sunset date of October 31, 2020, for the expiration of the 2014 Farm Bill language was extended twice, first to September 30, 2021, and then to January 1, 2022

While the 2018 Farm Bill sought to clarify the program framework and address inconsistencies resulting from the rulemaking process for newly legalized hemp, this has been complicated by the 2014 Farm Bill still being active related to hemp provision. With two Farm Bills being active simultaneously, an unlevel playing field is created as sampling, testing procedures, harvest windows, and testing frequency vary by state (Mark et al. 2020). In addition, the original sunset date of October 31, 2020, for the 2014 Farm Bill language for hemp has been extended twice, first to September 30, 2021, and then to January 1, 2022 (Fig. 1). State governments that passed hemp legislation prior to the 2018 Farm Bill have continued to regulate hemp production under the 2014 Farm Bill, while those who passed legislation in 2018 and later regulated according to the 2018 Farm Bill. As a result, this infant industry is now trying to overcome regulatory hurdles from two different Farm Bills that are not consistent.

It is important to note that these state and tribal hemp production plans only address the process of growing hemp up through pre-harvest THC compliance testing. After harvest (from the farm gate to retail product), regulations for hemp are also state and tribal government dependent but are not addressed by any of these plans. There are no federal-level retail regulations or industry standards that regulate the final hemp-based products in terms of consistency, quality, or analysis of claimed attributes of a hemp product in the retail sector. However, the US Food and Drug Administration (FDA) has launched a Data Acceleration Plan to learn more about the safety of cannabis-derived products, indicating that regulations for this sector are in progress (US Food and Drug Administration 2021).

Since the plans analyzed in this study only address hemp production up to harvest, inconsistencies between regulations have varying effects on the development of the industry, which may be compounded throughout the supply chain. For example, in the period between official testing and harvest, the plant continues to mature. As the plant matures, the THC level continues to increase and can result in a hemp plant that pushes its THC content over the allowable threshold (Pearce et al. 2021). This results in a producer having received a legal certificate of analysis from the appropriate authorities to harvest what was hemp at the time of testing but is legally marijuana at the time of harvest. Suppose testing is done post-harvest by a processing facility or is tested entering another state. In that case, serious issues can arise and result in the confiscation of the crop because of its illegal THC content (Pearce et al. 2021). As highlighted by this example, the lack of consistency between current regulations and the absence of successive regulations can impact the intra- and interstate flow of hemp and THC performance testing requirements, licensing fees, capital investment, and many other aspects of the industry.

Given the current lack of consensus regarding hemp production legislation, the objective of this study is twofold. First, the research team performed a content analysis to examine the consistency of terms between state and tribal hemp plans. Second, the study’s findings are used to provide recommendations to US governing bodies on how to improve clarity for hemp producers, thus mitigating regulatory confusion impeding the industry’s success. The format for the remainder of this manuscript is a review of methods for the content analysis, results, and discussion and conclusions.

## Methods

To analyze the consistency of terms between state and tribal hemp plans, the full narratives of each plan needed to be thoroughly examined and recorded. Once this was completed, a content analysis was used to translate the information provided by approved state and tribal government hemp plans into quantitative data. A similar content analysis approach was used in an analysis of sub-national insect pollinator legislation by Hall and Steiner (Hall and Steiner 2021), where content analysis allowed for both quantitative and qualitative descriptions of US policy. As defined by Krippendorff, content analysis is “a research technique for making replicable and valid inferences from texts to the contents of their use” ( (Krippendoff 2018) p. 24). In addition, the content analysis provides a systematic approach for quantifying and describing specific aspects of qualitative data (Downe-Wamboldt 1992). Originating in journalism, content analysis has grown in popularity and is used throughout varying disciplines, including business, communication, sociology, and medicine (Krippendoff 2018; Downe-Wamboldt 1992; Neuendorf 2016). Once the information from the hemp plans was translated into its quantitative form, the data were then used to identify common and idiosyncratic uses of terms and their definitions.

The cutoff date for this analysis was July 14, 2021. At that point, 67 states and tribal governments had approved independent plans, six were operating under the USDA Hemp Producer License, 20 were continuing to operate under the 2014 pilot, two were drafting a plan for USDA review, seven were under review, and two were pending legislation (Table 1) (U.S. Department of Agriculture Agricultural Marketing Service 2021). This study analyzed 69 state and tribal hemp production plans found on the official USDA Agricultural Marketing Service webpage (U.S. Department of Agriculture Agricultural Marketing Service 2021) including approved independent plans, the 2014 pilot plan, and the USDA Hemp Producer License. States electing to operate under the 2014 pilot plan were not assessed individually. Instead, the common pilot plan was analyzed and counted as one plan in the final plan count. The same approach was applied to plans operating under the USDA Hemp Producer License. As mentioned above, the 2014 pilot plan is not representative of the USDA Hemp Producer License. For this reason, we included both the 2014 pilot plan and USDA Hemp Producer License to evaluate the consistency between the two.

**Table 1:** Status of state and tribal government plans as of July 14, 2021

Plan status	Tribal governments	States
Independent Approved Plan	Blackfeet Nation Tribal Council, Cayuga, Cheyenne and Arapaho Tribe, Cheyenne River Sioux, Chippewa Cree, Colorado River Indian Tribes, Comanche Nation, Confederated Tribes of Warm Springs, Cow Creek Band of Umpqua Tribe of Indians, Eastern Band of Cherokee Indians, Flandreau Santee Sioux, Fort Belknap Indian Community, Iowa Tribe of Kansas and Nebraska, La Jolla Band of Luiseno Indians, Lac Courte Oreilles, Little Traverse Bay Bands of Odawa Indians Waganakising Odawak, Lower Sioux Indian Community, Miccosukee Tribe of Indians of Florida, Nez Perce Tribe, Oglala Sioux Tribe, Otoe-Missouria Tribe, Pala Band of Mission Indians, Pawnee Nation of Oklahoma, Prairie Band Potawatomi Nation, Pueblo of Picuris Tribe, Red Lake Band of Chippewa, Rosebud Sioux Tribe, Sac & Fox Tribe of the Mississippi in Iowa, San Carlos Apache Tribe of Arizona, Santa Rosa Band of Cahuilla Indians, Santee Sioux Nation, Seminole Nation of Oklahoma, Seneca Nation, Sisseton-Wahpeton Oyate, Soboba Band of Luiseno Indians, Standing Rock Sioux Tribe, Torres Martinez Desert Cahuilla Indians, Turtle Mt. Band of Chippewa Indians, Winnebago Tribe of Nebraska, Ysleta Del Sur Pueblo, Yurok Tribe	Delaware, Florida, Georgia, Indiana, Iowa, Kansas, Louisiana, Maryland, Massachusetts, Michigan, Minnesota, Missouri, Nebraska, Nevada, New Jersey, Ohio, Oklahoma, Pennsylvania, Puerto Rico, Rhode Island, South Carolina, South Dakota, Texas, U.S. Virgin Islands, Washington, Wyoming,
Operating Under USDA Hemp Producer License	Assiniboine and Sioux Tribes of the Fort Peck Reservation, Confederated Salish & Kootenai Tribes of the Flathead Reservation, Lower Brule Sioux Tribe	Hawaii, Mississippi, New Hampshire
Continuing Under 2014 Pilot	—	Alabama, Alaska, Arkansas, Colorado, Connecticut, Illinois, Kentucky, Maine, Montana, New Mexico, New York, North Carolina, North Dakota, Oregon, Tennessee, Utah, Vermont, Virginia, West Virginia, Wisconsin
Drafting Plan for USDA Review	Ute Mountain Ute, Yankton Sioux Tribe	—
Under Review	Cahuilla Band of Indians, Kanosh Band of Paiute Indians, Pauma Band of Luiseno Indians, Saint Regis Mohawk Tribe, Shoshone-Bannock Tribes	Arizona, California,
Pending Legislation	—	Idaho, Northern Marianas Island
2014 Pilot Plan	—	—
USDA Hemp Producer License	—	—

Adapted from: US Department of Agriculture Agricultural Marketing Service, 2021. All plan statuses are as of July 14, 2021. Independent Approved Plans: plans submitted by states, tribes, or territories that have been approved by the USDA. USDA Hemp Producer License: plans for producers in states, tribes, or territories that have elected to follow USDA guidelines. Continuing Under 2014 Pilot: states, tribes, or territories choosing to continue their hemp production using the 2014 pilot plan. For current status and details of all plans, go to https://www.ams.usda.gov/rules-regulations/hemp/licensed-producers

Researchers from the Universities of Vermont and Kentucky conducted a preliminary analysis of all approved state and tribal plans. This study used three human coders (i.e., *team*), to ensure coding consistency and accelerate data coding efficiently once intercoder reliability was assured. While intercoder reliability is essential to establish whenever research involves more than one coder, this is especially true when quantifying qualitative data (Burla et al. 2008). To begin, the team documented all terms and definitions included in the 69 plans. The team was comprised of three research assistants. All three research assistants meet weekly to review work, discuss discrepancies, and adapt the coding scheme as needed to insure consistency among the coders (Krippendoff 2018). Table S1 within the Supplemental Materials contains the form utilized by all coders to collect the required information. A second coder completed a subsequent round of this step to ensure that all terms had been identified. During these rounds of analysis, coders identified terms they felt were “common knowledge.” Example “common knowledge” terms included but are not limited to negligent, consumer, laboratory, greenhouse, outdoor grow, etc. If a term that had initially been identified as “common knowledge” by one coder was considered necessary to include by another, the coders revisited the plans to ensure that the term was included in the final list of terms for the analysis. Examples of the terms not included due to their “common knowledge” designation are GPS, laboratory, USDA, and secretary. The coding team determined that these terms had definitions well-known by the public and did not provide any added information specific to hemp regulations. These terms were not included in the formal analysis.

**Fig. 2:**
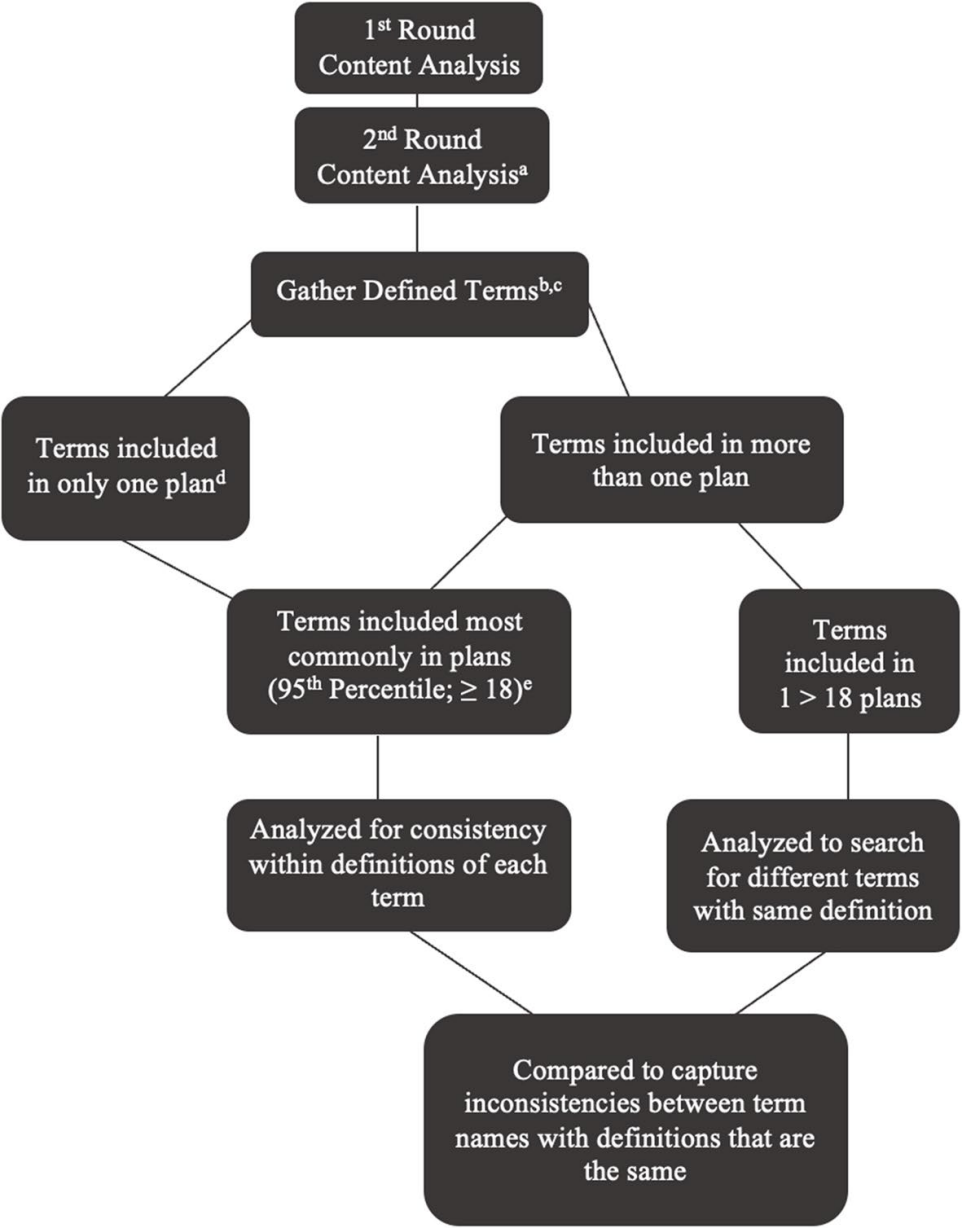
Schematic of content analysis methods. ^a^69 total plans were evaluated. ^b^*n* = 421. ^c^*n* does not include terms that were deemed “common knowledge.” ^d^241 terms were included in only one plan. ^e^24 terms were included in 18 or more plans. This schematic provides a visualization of the content analysis process used for this manuscript

**Fig. 3:**
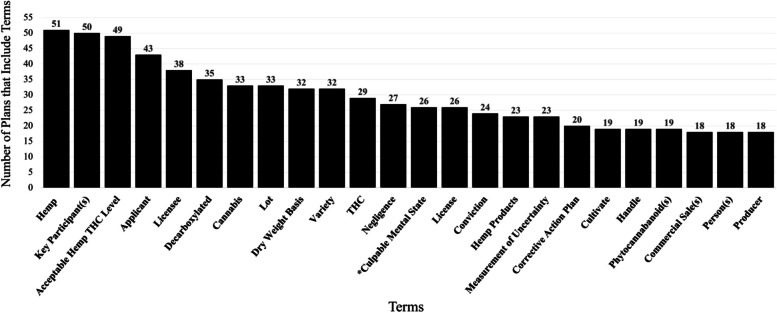
Number of plans that include most common terms. Total number of plans to include the most common terms (those mentioned 18 or more times). *N* = 69. *Full term name is “culpable mental state greater than negligence”

Due to the large number of terms included across plans, the research team took two steps to establish inter-plan consistency. Figure 2 details each step of the content analysis completed for this study. First, the team established which terms were most frequently used by employing percentile ranks. For this part of the analysis, terms deemed “most frequently” included were those which were in 18 or more plans, based on the number of plans to include a term (of the 69 analyzed). The research group then scrutinized definitions provided for each term to determine if consistent definitions were given throughout all plans that included the term. Intercoder reliability was achieved for this part of the analysis by requiring all three coders to review and agree upon the consistency of the definitions provided by plans. This study discounted slight variations in verbiage when determining whether the definitions were consistent. To provide a percentage of definition consistency across plans, the number of times a definition was provided for each term was divided by the total number of plans that included the term. For example, the term “Hemp” occurred in 51 of the 69 plans (73.91%), and the most common definition appeared in 37 of the 51 plans (72.55%) (Fig. 3; Table 2).

**Table 2:** Consistency of terms and definitions used in 69 US state and tribal hemp production plans

Term	Percentage of plans that include term	Percentage of plans that include most common definition	Most common definition
Hemp	73.9	72.6	“The plant *Cannabis sativa L*. and any part of that plant, including the seeds thereof and all derivatives, extracts, cannabinoids, isomers, acids, salts, and salts of isomers, whether growing or not, with a delta-9 THC concentration of not more than 0.3 percent on a dry weight basis.”
Key Participant(s)	72.45	80.0	“A sole proprietor, a partner in partnership, or a person with executive managerial control in a corporation. A person with executive managerial control includes persons such as a chief executive officer, chief operating officer and chief financial officer. This definition does not include non-executive managers such as farm, field, or shift managers.”
Acceptable Hemp THC Level	71.0	67.4	“When a laboratory tests a sample, it must report the delta-9 tetrahydrocannabinol content concentration level on a dry weight basis and the measurement of uncertainty. The acceptable hemp THC level for the purpose of compliance with the requirements of the Tribe's hemp plan is when the application of the measurement of uncertainty to the reported delta-9 tetrahydrocannabinol content concentration level on a dry weight basis produces a distribution or range that includes 0.3% or less. For example, if the reported delta-9 tetrahydrocannabinol content concentration level on a dry weight basis is 0.35% and the measurement of uncertainty is ± 0.06%, the measured delta-9 tetrahydrocannabinol content concentration level on a dry weight basis for this sample ranges from 0.29% to 0.41%. Because 0.3% is within the distribution or range, the sample is within the acceptable hemp THC level for the purpose of plan compliance. This definition of "acceptable hemp THC level" is not meant to affect either the statutory definition of hemp in the 2018 Farm Bill (codified at 7 U.S.C. § 16,390(1)) or the definition of "marihuana" in the Controlled Substances Act (codified at 21 u.s.c. § 802(16)).”
Applicant	62.3	74.4	“A person, or a person who is authorized to sign for a business entity, who submits an application to participate in the Hemp program.”
Licensee	55.1	0	N/A
Decarboxylated	50.7	54.3	“The completion of the chemical reaction that converts THC-acid (“THC- A”) into delta-9-THC, the intoxicating component of cannabis. The decarboxylated value is also calculated using a conversion formula that sums delta-9-THC and eighty-seven and seven tenths (87.7) percent of THC-A.”
Cannabis	47.8	69.7	“A genus of flowering plants in the family Cannabaceae of which *Cannabis sativa* is a species, and Cannabis indica and Cannabis ruderalis are subspecies thereof. Cannabis refers to any form of the plant in which the delta-9 tetrahydrocannabinol concentration on a dry weight basis has not yet been determined.”
Lot	47.8	39.4	“A contiguous area in a field, greenhouse, or indoor growing structure containing the same variety or strain of cannabis throughout the area.”
Dry Weight Basis	46.34	50.0	"The ratio of the amount of moisture in a sample to the amount of dry solid in a sample. A basis for expressing the percentage of a chemical in a substance after removing the moisture from the substance. Percentage of THC on a dry-weight basis means the percentage of THC, by weight, in a cannabis item (plant, extract, or other derivative), after excluding moisture from the item.”
Variety	46.4	46.9	“A subdivision of a species that is uniform, in the sense that the variations in essential and distinctive characteristics are describable, stable, in the sense that the variety will remain unchanged in its essential and distinctive characteristics and its uniformity if reproduced or reconstituted as required by the different categories of varieties, and distinct, in the sense that the variety can be differentiated by one or more identifiable morphological, physiological, other characteristics from all other publicly known varieties, or other characteristics from all other publicly known varieties.”
THC	42.0	27.6	“Tetrahydrocannabinol and has the same meaning as delta-9 THC, measured post-decarboxylation.”
Negligence	39.1	96.3	“A failure to exercise the level of care that a reasonably prudent person would exercise in complying with this Plan.”
Culpable Mental State Greater Than Negligence	37.7	50.0	“To act intentionally, knowingly, willfully, or recklessly.”
License	37.7	0	N/A
Conviction	34.8	79.2	“Any plea of guilty or nolo contendere, or any finding of guilt, except when the finding of guilt is subsequently overturned on appeal, pardoned, or expunged. For purposes of this Plan a Conviction is expunged when the Conviction is removed from the individual’s criminal history report and there are no legal disabilities or restrictions associated with the expunged Conviction, other than the fact that the Conviction may be used for sentencing purposes for subsequent Convictions. When an individual is allowed to withdraw an original plea of guilty or nolo contender and enter a plea of not guilty and the case is subsequently dismissed, the individual is no longer considered to have a Conviction for purposes of this Plan.”
Hemp Product(s)	33.3	30.4	“Means a finished product with the Acceptable Hemp THC Level that is derived from, or made by, processing a Hemp Crop, and that is prepared in a form available for commercial sale. The term includes, but is not limited to cosmetics, personal care products, Consumable Products, cloth, cordage, fiber, fuel, paint, paper, particleboard, plastics, and any product containing one or more Hemp Ingredients such as cannabidiol.”
Measurement of Uncertainty	33.3	87.0	“The parameter, associated with the result of a measurement, that characterizes the dispersion of the values that could reasonably be attributed to the particular quantity subject to measurement.”
Corrective Action Plan	29.0	75.0	“Means a plan for a licensed hemp producer to correct a negligent violation or non-compliance with a hemp production plan and this program.”
Cultivate	27.5	84.2	“To plant, water, grow, and harvest a plant or crop.”
Handle	27.5	31.6	“To harvest or store hemp or hemp plant parts prior to the delivery of such plants or plant parts for further processing. "Handle" also includes the disposal of cannabis plants that are not hemp for the purposes of chemical analysis and disposal of such plants.”
Phytocannabanoid(s)	27.5	89.5	“Cannabinoid chemical compounds found in the cannabis plant, two of which are Delta-9 tetrahydrocannabinol (delta-9 THC) and cannabidiol (CBD).”
Commercial Sale(s)	26.1	100.0	“The sale of a product in the stream of commerce at retail or at wholesale, including sales on the internet.”
Person(s)	26.1	27.8	“A natural person, corporation, foundation, organization, business trust, estate, limited liability company, licensed corporation, trust, partnership, limited liability partnership, association, or other form of legal business entity, as well as a tribal, state or local government entity.”
Producer	26.1	44.4	“An owner, operator, landlord, tenant, or sharecropper, who shares in the risk of producing a crop and who is entitled to share in the crop available for marketing from the farm or would have shared had the crop been produced. A producer includes a grower of hybrid seed.”

*N* = 69. For this part of the analysis, terms deemed “most frequently” included were those which were mentioned in 18 or more of the 69 plans analyzed. The research group analyzed definitions provided for each term to determine if consistent definitions were given throughout all plans that included the term. Slight variation in verbiage was disregarded when determining the most common definition. To calculate the values for the “percent of plans that include term,” the number of plans each term appeared in was divided by 69 (the total number of plans analyzed). To calculate the percentage of definition consistency across plans, the number of times a definition was provided for each term was divided by the total number of plans that included the term

Terms included between two and 17 times were reviewed for consistency between definitions across terms with different names (Fig. 2). As with the first consistency analysis described above, this study disregarded slight variations in verbiage when determining whether the definitions were the same. Intercoder reliability was ensured for this step by requiring all group members to sort through the terms which occurred between two and 17 times to identify those which fit this criterion. Similarly defined terms with different names were grouped based on the content of their definitions. Terms that did not fit this criterion were not analyzed further. Additionally, terms that were included in only one plan were not analyzed further.

## Results

In the 69 plans analyzed, 421 different terms were identified. Twenty-four terms were included in 18 or more plans (Fig. 3). The term most frequently cited in plans was “hemp,” which appeared in 51 of the 69 plans. The research team found substantial variation in term definitions across plans, with inter-plan consistency ranging from 0 to 100% (Table 2). Only one term, “commercial sales,” was defined consistently across all plans that included the term, while “License” and “Licensee” had no consistency between definitions across plans. All definitions for these two terms refer to the same concept, yet the wording varied drastically enough to be deemed inconsistent.

The terms “THC” and “Hemp Product” were defined consistently in 30% of plans. The terms “Lot,” “Variety,” and “Producer” were defined consistently in fewer than 50% of plans. “Culpable Mental State Greater Than Negligence” and “Dry Weight Basis” were defined consistently in 50% of the plans. “Acceptable Hemp THC Level” was defined consistently in fewer than 70% of plans. “Hemp,” “Applicant,” “Cannabis,” and “Corrective Action Plan” were consistently defined in 75% of plans. Definitions of “Key participant,” “Negligence,” “Cultivate,” “Measurement of Uncertainty,” and “Negligence” were consistent in more than 80% of plans. Table 2 shows the analyzed terms, the percentage of total plans that the term appeared in, and the percentage of those plans that use the most common definition to appear throughout all plans.

**Fig. 4:**
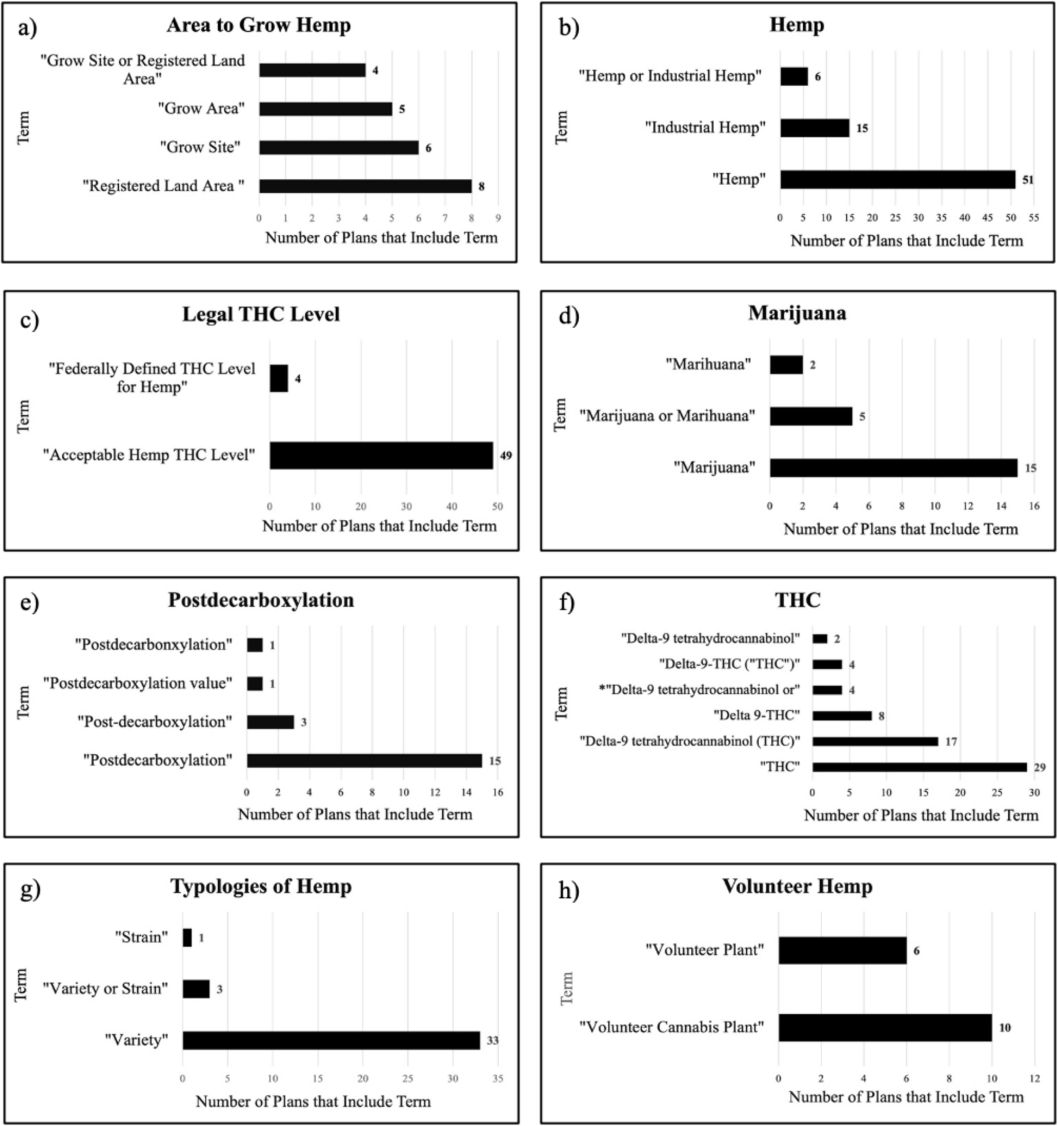
Terms grouped by similar definition content. Term groupings based on definition similarity. Each subfigure signifies one of the eight groups identified. Slight variation in definition verbiage was disregarded when determining similarities between definitions. **a** Terms defining areas to grow hemp. **b** Terms defining hemp. **c** Terms defining the legal THC level. **d** terms defining marijuana. **e** Terms defining the process of postdecarboxylation. **f** terms defining THC. Full term name is *Delta-9-tetrahydrocannabinol or THC or Delta-9-THC. **g** Terms defining different typologies of hemp. **h** Terms defining volunteer hemp

Terms that appear more than once but did not meet the 18 plan threshold were analyzed further. For this analysis, terms that had the same or similar definitions, but different names, were grouped together and categorized by the research team. As with the other consistency analysis, slight variation in verbiage was disregarded when determining whether the definitions were the same. The eight groups that were identified were “Area to Grow Hemp,” “Hemp,” “Legal THC Level,” “Marijuana,” “Postdecarboxylation,” “THC,” “Typologies of Hemp,” and “Volunteer Hemp” (Fig. 4). All terms listed within each group were described using the same or very similar definitions. For example, in the “Hemp” category, the terms “Hemp or Industrial Hemp,” “Industrial Hemp,” and “Hemp” are listed, meaning that the definitions for these terms all define hemp. The least common terms were included in the analyzed plans only once. Of the 421 total terms identified for analysis, 241 (57.24%) were only included in only one plan. The complete list of included terms included in the analysis, as well as the number of plans they occurred in, can be found in Table S2 of the Supplementary Material.

## Discussion

When beginning this study, the research team expected to discover that the introduction of the final rule on March 21, 2021, would provide relatively more consistency across state and tribal government plans than was seen after the deployment of the 2014 pilot plans. However, it appears that providing each state and tribal government the opportunity to submit an independent plan for approval has done the opposite. This is emphasized by the 241 terms included only in one plan. Furthermore, inconsistent term names were provided for the same definition among the 180 terms that appear between two and 17 times throughout the 69 plans analyzed. Lastly, 23 of the 24 terms that were included most frequently across plans (included in ≥ 18 plans) were given incongruous definitions, demonstrating different understandings of the term. The findings of this study highlight the persistent inconsistencies in hemp production regulations among US states and tribal governments.

While the varying terminology in state and tribal plans is likely due to different colloquialisms across the country, these disparities can create regulatory confusion. Since hemp is highly likely to be transported and marketed across state and tribal boundaries, differences in regulatory language at such an early stage create challenges for actors throughout the supply chain, including producers, input suppliers, processors, marketers, and consumers. In addition, inconsistencies limit future expansion by creating additional barriers such as new market entry, customer loyalty and acquisition of new and valuable venture capital (Mark et al. 2020). Furthermore, the inaccurate association between hemp and cannabis has proven to be a barrier to success for industrial hemp production for decades. Yet, despite this differentiation, the distinction between the two plants is not easily discerned by members of the American public, with one 2020 study of Southeastern United States residents reporting 29% of respondents associating hemp with recreational marijuana (Williams et al. 2020).

It is important to note that while our team has concluded that the independent state and tribal hemp plans are noticeably inconsistent, the presence of this varying terminology is not indicative of a true discrepancy in the production of hemp between these entities. Without being familiar with the intricacies of each hemp production plan, we are unable to say for certain the degree to which the practices of each entity differ. However, the findings of this study suggest that there is likely some discordance between hemp production in each state and tribal government that has an approved independent plan.

As highlighted by its tumultuous history, a major barrier to the success of the reintroduction of hemp in the US agricultural sector is its association with cannabis (Campbell et al. 2021; Williams et al. 2020). If we suppose the objective of federally approved hemp production plans is to mitigate the ability of hemp producers to abuse their license to grow *Cannabis sativa L.* and cultivate cannabis instead of hemp, it is reasonable to believe that the incongruent composition of state and tribal plans makes this challenging to prevent. By allowing states and plans to determine different windows for post-test harvest, for example, the current regulations may unintentionally allow for the distribution of cannabis (Pearce et al. 2021). This presents several threats to the success of hemp: notably the confusion of consumers and inability to engage in interstate commerce. Therefore, ensuring that the two plants remain separate crops will be integral to the prosperity of the hemp market.

Findings from this study point to areas in need of uniformity and consistency as the regulatory framework is modified and provides a starting point for federal policymakers. Based on the conclusions of our analysis, it appears that current regulatory flexibility has created an environment that fosters competitive advantages among state and tribal governments depending upon the content of their independent plans. However, more research is needed to fully understand the scope and depth of any potential competitive advantage. Further, the democratic process will have to play out as states and tribal governments will most likely be interested in maintaining any current advantage, whether intentional or unintentional. Lastly, it is likely that states and tribal governments have had newly developed and approved hemp production plans since the cutoff date for this analysis, July 14, 2021. As a result, there is an opportunity for further research to analyze if these changes have enhanced, or further weakened, the overall consistency of plans.

## Conclusions

Based on the findings of this study, there are significant areas for improvement in federal policy guidelines for hemp production. The research team has curated two suggestions for how to mitigate the inconsistencies seen in state and tribal hemp programs. First, we recommend that the USDA provide and define the basic regulatory language for independent plans to follow. While the USDA Hemp Producer License provides some terminologies and definitions, it is not required that plans choosing to operate under individually approved plans adhere to them. By creating an expanded list of terms and corresponding definitions that must be ubiquitous among all state and tribal plans, the USDA can provide a lexicon for hemp producers to alleviate discrepancies in how production is approached and defined.

Additionally, we suggest the creation of regulations for the rest of the hemp supply chain. While we are aware that the USDA does not have jurisdiction over the processing of hemp or any other steps post-harvest, we feel that it will be beneficial to provide these regulations to ensure that, once cleared on the pre-harvest side, the integrity of the hemp programs is maintained and are not allowed to infiltrate the cannabis business. By creating clear separations between hemp and cannabis supply chains, hemp producers may find relief from the longstanding erroneous association between the two crops. For the USA to steward a victorious reemergence of hemp in the agricultural sector, the industry must work with policymakers and regulators to attenuate pre-existing barriers and provide a way for hemp to safely and equitably make its way to consumers who have confidence in purchasing various hemp-derived products.

## Supplementary Information

**Additional file 1: Table S1.** Form used to document term, definition, and state/tribal government. Description of Data: Example format of the form used to document the term, the corresponding definition, and which state or tribal government’s plan the term was found in. **Table S2.** List of terms included in only one plan. Description of Data: A comprehensive list of the terms included in only one plan at the research study cutoff date of July 14, 2021.

## Data Availability

The datasets used and/or analyzed during the current study are available from the corresponding author on reasonable request.

## Abbreviations

USDA: United States Department of Agriculture
THC: Δ^9^-Tetrahydrocannabinol
FDA: Food and Drug Administration
TEAM: Coding team

## References

[CR1] Agricultural Act 2018. Washington: House of Representatives 2 115^th^ Congress §7605. Available from: https://www.congress.gov/115/bills/hr2/BILLS-115hr2enr.pdf. cited 2021 Aug 2

[CR2] Agricultural Act 2014. Washington: House of Representatives 2642 113^th^ Congress § 7606. Available from: https://www.govinfo.gov/content/pkg/BILLS-113hr2642enr/pdf/BILLS-113hr2642enr.pdf. cited 2021 Aug 2

[CR3] Burla L, Knierim B, Barth J, Liewald K, Duetz M, Abel T (2008). From text to codings: intercoder reliability assessment in qualitative content analysis. Nurs Res..

[CR4] Campbell B, Mark T, McFadden B, Rabinowitz A (2021). Reporting survey data from February through April (first quarter of data collection).

[CR5] Downe-Wamboldt B (1992). Content analysis: method, applications, and issues. Health Care Women Intl..

[CR6] Hall DM, Steiner R. Policy content analysis: qualitative method for analyzing sub-national insect pollinator legislation.MethodsX. 2020;13. 10.1016/j.mex.2020.100787 cited 2021 Sep 19 10.1016/j.mex.2020.100787 PMC6996007 32025507

[CR7] Johnson R (2018). Hemp as an agricultural commodity.

[CR8] Johnson R (2019). Defining hemp: a fact sheet.

[CR9] Krippendoff K (2018). Content analysis: an introduction to its methodology [e-book].

[CR10] Malone T, Gomez K (2019). Hemp in the United States: a case study of regulatory path dependence. Appl Econ Perspect Policy..

[CR11] Mark T, Shepherd J, Olson D, Snell W, Proper S, Thornsbury S (2020). Economic viability of industrial hemp in the United States: a review of state pilot programs.

[CR12] Neuendorf KA (2016). 2020. The content analysis guidebook [e-book].

[CR13] Pal L, Lucia LA (2019). Renaissance of industrial hemp: a miracle crop for a multitude of products. BioResources..

[CR14] Pearce B, Valentine T, Keene T, Sikora F, Hamilton D. Cannabinoid accumulation in floral hemp cultivars: implications for harvest management*.* [PowerPoint presentation]. Kentucky Department of Agriculture, University of Kentucky. 2021 [cited 2021 Nov 30].

[CR15] United States Department of Agriculture Economic Research Service (2000). Industrial hemp in the United States: status and market potential.

[CR16] U.S. Department of Agriculture Agricultural Marketing Service (2021). Hemp production.

[CR17] U.S. Department of Agriculture Agricultural Marketing Service (2021). Status of state and tribal hemp production plans for USDA approval.

[CR18] U.S Drug Enforcement Administration (2020). Marijuana/cannabis drug fact sheet.

[CR19] US Food and Drug Administration (2021). Cannabis-derived products data acceleration plan.

[CR20] Williams J, Campbell J, Campbell B, Rabinowitz A, Campbell J (2020). Consumer views on use and legality of hemp based products.

