# Supplementary material for: The reintroduction of hemp in the USA: a content analysis of state and tribal hemp production plans
Source: J Cannabis Res. 2023 Jun 7;5:17. doi: 10.1186/s42238-023-00181-0 (PMC10245984; doi:10.1186/s42238-023-00181-0)
Supplement: Supplementary file 1 — Additional file 1: Table S1. Form used to document term, definition, and state/tribal government. Description of Data: Example format of the form used to document the term, the corresponding definition, and which state or tribal government’s plan the term was found in. Table S2. List of terms included in only one plan. Description of Data: A comprehensive list of the terms included in only one plan at the research study cutoff date of July 14, 2021. [file 42238_2023_181_MOESM1_ESM.docx]

**Table 1. Form used to document term, definition, and state/tribal government**

| Term | Definition | State/Tribal Government |
| --- | --- | --- |
|  |  |  |
|  |  |  |
|  |  |  |

A replication of the excel template used to track all terms, the corresponding definitions, and which state or tribal entity’s plan it was included in.

**Table 2.** Comprehensive list of terms included in analysis

| Term | Number of Plans to Include Term |
| --- | --- |
| Acceptable Hemp THC Level | 49 |
| Acceptable Industrial Hemp THC Level | 1 |
| Acceptable THC Level | 5 |
| Administrative License | 1 |
| Adulterated | 1 |
| Agent | 1 |
| Agricultural Hemp Propagule (Propagule) | 1 |
| Agricultural Hemp Propagule and Seed Permit (Permit) | 1 |
| Agricultural Hemp Seed | 2 |
| Agricultural Hemp Seed (Seed) | 1 |
| Agricultural Pilot Program | 1 |
| Agriculture Office | 2 |
| Applicant | 43 |
| Application | 3 |
| Approved Seed | 1 |
| Approved Variety of Industrial Hemp | 1 |
| Authorized Agent | 1 |
| Authorized Laboratory | 1 |
| Batch | 1 |
| Bonafide Farmer Certificate | 1 |
| Broker | 3 |
| Brokering | 1 |
| Building | 1 |
| Burning | 1 |
| Bush Mower/Chopper | 1 |
| Cannabidiol or CBD | 6 |
| Cannabinoid Profile | 2 |
| Cannabinoid(s) | 4 |
| Cannabinol (CBN) | 1 |
| Cannabis | 33 |
| Cannabis Sativa L | 1 |
| CBD | 15 |
| CBD Biomass | 1 |
| CBD Broad Spectrum Oil Distillate | 1 |
| CBD Full Spectrum Oil Distillate | 1 |
| CBD Seeds (Non-Feminized) | 1 |
| CBD/CBG Clones | 1 |
| CBD/CBG Isolate | 1 |
| CBD/CBG Seeds (Feminized) | 1 |
| CBDA | 1 |
| CBG Biomass | 1 |
| CBG Distillate | 1 |
| Certificate | 2 |
| Certificate of Analysis (“COA”) | 3 |
| Certified Hemp Seed | 1 |
| Certified Industrial Hemp Sampler (Certified Sampler) | 1 |
| Certified or Approved Hemp Seed | 1 |
| Certified Seed | 7 |
| Certified Seed/Low THC Seed | 1 |
| Certifying Agency | 1 |
| Commercial | 2 |
| Commercial Sale(s) | 18 |
| Commission | 1 |
| Commissioner | 4 |
| Compliance Transaction | 1 |
| Compliant Hemp | 1 |
| Compliant Industrial Hemp | 1 |
| Condition | 1 |
| Consumable | 1 |
| Consumable Hemp Product | 1 |
| Consumable Product | 10 |
| Consumer | 1 |
| Container | 1 |
| Contiguous | 2 |
| Contiguous Field | 3 |
| Contiguous Land Area | 1 |
| Contiguous Licensing | 1 |
| Control Order | 1 |
| Controlled Substance | 1 |
| Controlled Substance Felony | 1 |
| Controlled Substances Act | 4 |
| Conviction | 24 |
| Corrective Action Plan | 20 |
| Corrective Action Plan or CAP | 1 |
| Criminal History Record Information | 1 |
| Criminal History Report | 12 |
| Crop | 3 |
| Crop Destruction | 1 |
| Crop Site | 1 |
| Crop Termination | 1 |
| Crude Hemp Oil | 1 |
| Culpable Mental State Greater Than Negligence | 26 |
| Cultivar | 2 |
| Cultivate | 19 |
| Cultivate or Cultivating | 2 |
| Cultivating | 2 |
| Cultivating or Cultivation | 4 |
| Cultivation | 2 |
| Cultivation License | 1 |
| Cultivation Licensing Agreement | 1 |
| Cultivation Site | 4 |
| Cultivator | 1 |
| Date of Harvest | 1 |
| DEA Registered Reverse Distributor or a Duly Authorized Federal, State, or Local Law Enforcement Officer | 1 |
| Decarboxylated | 35 |
| Decarboxylation | 17 |
| Deep Burial | 1 |
| Delta 9-THC | 8 |
| Delta-9 tetrahydrocannabinol | 2 |
| Delta-9 tetrahydrocannabinol (THC) | 17 |
| Delta-9 tetrahydrocannabinol or THC or Delta-9-THC | 4 |
| Delta-9 THC Post-Decarboxylation | 1 |
| Delta-9-THC ("THC") | 4 |
| Delta-9-THCA ("THC-A") | 1 |
| Designated Responsible Party | 1 |
| Destroy | 1 |
| Destroy(ed) | 1 |
| Destruction Report | 1 |
| Destruction/Disposal | 4 |
| Devitalize | 1 |
| Disking | 1 |
| Disposal | 1 |
| Disqualifying Felony | 1 |
| Distribute/Distribution | 2 |
| Dried CBD Flower | 1 |
| Drug Felony Conviction Report | 1 |
| Dry Weight Basis | 32 |
| Dwelling | 1 |
| Entity | 10 |
| Establishment | 1 |
| Extractor | 1 |
| Extractor or Extraction | 1 |
| Facility | 2 |
| Familial Interest | 1 |
| Federally Defined THC Level for Hemp | 4 |
| Federally Defined THC Level for Hemp or Acceptable Hemp THC Level | 1 |
| Field | 2 |
| Field Average | 1 |
| Field Duplicate Sample | 1 |
| Final Sample | 2 |
| Final Test | 2 |
| Financial Interest | 6 |
| Fit for Commerce | 1 |
| Flowering Plant | 1 |
| Fund | 1 |
| Gas Chromatography | 6 |
| Gas Chromatography and High-Performance Liquid Chromatography | 1 |
| Gas Chromatography or GC | 12 |
| Gas or Liquid Chromatography with Detection | 1 |
| General Permit | 1 |
| Genuine Grower’s Declaration | 1 |
| Governing Person | 1 |
| Grain | 1 |
| Greenhouse | 3 |
| Ground Cover | 1 |
| Grow | 1 |
| Grow Location | 1 |
| Grow or Growing | 1 |
| Grow Site | 6 |
| Grow Site or Registered Land Area | 4 |
| Grower | 10 |
| Grower Licensing Agreement | 1 |
| Growing Area | 5 |
| Growing Area or Site | 1 |
| Growing Location or Lot | 1 |
| Growing Plant | 1 |
| Guarantor | 1 |
| Handle | 19 |
| Handle or Handling | 7 |
| Handler | 10 |
| Handling | 7 |
| Handling Site | 1 |
| Harvest | 8 |
| Harvest Certificate | 1 |
| Harvest Form | 2 |
| Harvest Lot | 12 |
| Harvest Lot Identifier | 12 |
| Harvest Lot or Lot | 1 |
| Harvest/Destruction Report | 1 |
| Harvesting | 1 |
| HCO | 4 |
| Hemp | 51 |
| Hemp Activity | 2 |
| Hemp Bill of Lading | 1 |
| Hemp Business | 6 |
| Hemp Comission | 2 |
| Hemp Control Officer | 2 |
| Hemp Crop | 12 |
| Hemp Cultivation | 1 |
| Hemp Extract | 2 |
| Hemp Grower | 5 |
| Hemp Grower and Hemp Producer | 2 |
| Hemp Grower License or Grower License | 2 |
| Hemp Grower or Licensee | 2 |
| Hemp Handling Facility | 1 |
| Hemp Ingredient | 8 |
| Hemp Oil | 1 |
| Hemp or Industrial Hemp | 6 |
| Hemp Plan | 1 |
| Hemp Plant | 1 |
| Hemp Plant Parts | 1 |
| Hemp Processor | 5 |
| Hemp Processor Permit | 1 |
| Hemp Processor Permit/License or Processor Permit/License | 1 |
| Hemp Producer | 6 |
| Hemp Producer or Licensee | 1 |
| Hemp Product or Industrial Hemp Product | 1 |
| Hemp Product(s) | 23 |
| Hemp Production Site | 1 |
| Hemp Program | 3 |
| Hemp Propogative Material | 1 |
| Hemp Research License | 1 |
| Hemp Research Pilot Project Licenses | 1 |
| Hemp Researcher | 1 |
| Hemp Seller | 5 |
| Hemp Site | 1 |
| High Performance Liquid Chromatography or HPLC | 13 |
| High-performance Liquid Chromatography | 3 |
| High-performance Liquid Chromatography (HPLC) or (LC) | 1 |
| Immature plant | 1 |
| Individual | 1 |
| Indoor Crop Site | 1 |
| Indoor Cultivation | 1 |
| Indoor Production | 1 |
| Industrial Hemp | 15 |
| Industrial Hemp License or License | 1 |
| Industrial Hemp Plant Parts | 1 |
| Industrial Hemp Product(s) | 5 |
| Industrial Seeds | 1 |
| Intended for Consumption | 7 |
| Intended for Consumption or Consumable | 1 |
| Key Participant(s) | 50 |
| Laboratory License | 1 |
| Landowner | 1 |
| Legal Description | 1 |
| Lessee | 2 |
| License | 26 |
| License Agreement | 6 |
| License Application | 2 |
| License for the Importation and Distribution of Hemp Products for Consumption | 1 |
| License Holder | 1 |
| License Holder Who Transplants | 2 |
| Licensed Area | 3 |
| Licensed Cultivator | 1 |
| Licensed Grower | 5 |
| Licensed Growing area | 1 |
| Licensed Processor | 5 |
| Licensed Research Distributor | 1 |
| Licensed Research Grower | 1 |
| Licensed Research Processor | 1 |
| Licensed Research Section | 1 |
| Licensee | 38 |
| Licensee or Licensed Hemp Producer | 1 |
| Licensee or USDA Licensed Hemp Producer | 2 |
| Licensee Representative | 1 |
| Licensing Agreement | 1 |
| Listed Low THC seed | 1 |
| Location ID | 11 |
| Location or Land | 7 |
| Lot | 33 |
| Lot Identification | 1 |
| Manufacturer | 2 |
| Manufacturing License | 1 |
| Marihuana | 2 |
| Marijuana | 15 |
| Marijuana or Marihuana | 5 |
| Market or Marketing | 1 |
| Marketable Hemp Product | 2 |
| Material Change | 1 |
| Measure of Uncertainty | 1 |
| Measurement of Uncertainty | 22 |
| Measurement of Uncertainty or MU | 7 |
| Medical Cannabis | 1 |
| Mulching/Composting | 1 |
| Nebraska Heirloom Cannabis Plant or Seed | 1 |
| Negligence | 27 |
| Negligence or Negligent | 1 |
| Negligence, Negligent, Negligently | 4 |
| Negligent Violation | 3 |
| Non-commercial Personal Possession or Use | 2 |
| Non-compliant Hemp | 1 |
| Non-marketable Hemp | 1 |
| Non-retrievable | 2 |
| Nonconsumable Hemp Product | 2 |
| Nonviable Seed | 9 |
| Official Sample | 3 |
| Official Test Result | 1 |
| Outdoor Production | 1 |
| Parcel | 1 |
| Percentage of THC on a Dryweight Basis | 1 |
| Permit | 9 |
| Permit Holder | 1 |
| Permit or Lot Permit | 3 |
| Permitted Farmer or Permitted Hemp Farmer | 1 |
| Permitted Handler or Permitted Hemp Handler | 1 |
| Permitted Processor or Permitted Hemp Processor | 1 |
| Permittee | 3 |
| Person(s) | 18 |
| Person/s or Individuals | 1 |
| Personal Use | 1 |
| Pesticide | 12 |
| Phytocannabanoid(s) | 19 |
| Pilot Program | 1 |
| Pilot Project Hemp Cultivar | 1 |
| Pilot Project Hemp Seed | 1 |
| Plan | 1 |
| Plan/Program | 1 |
| Plant | 3 |
| Plant Health Office | 1 |
| Plant Part | 1 |
| Planting Form | 2 |
| Planting Report | 2 |
| Plantlets | 1 |
| Plot | 3 |
| Plot or Lot | 2 |
| Plowing Under | 1 |
| Possessor | 1 |
| Post-decarboxylation | 3 |
| Post-harvest Plant Material Waste | 1 |
| Post-harvest Sample | 2 |
| Postcarboxylation Test | 1 |
| Postdecarbonxylation | 1 |
| Postdecarboxylation | 15 |
| Postdecarboxylation Value | 1 |
| Postharvest Report | 1 |
| Pre-harvest Inspection | 1 |
| Pre-harvest Plant Material Waste | 1 |
| Pre-harvest Report | 1 |
| Pre-harvest Sample | 5 |
| Pre-harvest Testing | 1 |
| Primary Licensee | 1 |
| Process | 15 |
| Process or Processing | 9 |
| Processed Hemp Plant Material | 1 |
| Processing | 13 |
| Processing Area | 2 |
| Processing Locations | 1 |
| Processor License | 1 |
| Processor Licensing Agreement | 4 |
| Processor or Processor Facility | 6 |
| Processor-Handler | 1 |
| Processor(s) | 7 |
| Produce | 16 |
| Produce or Producing | 5 |
| Produce or Production | 2 |
| Producer | 18 |
| Producer Licensing Agreement | 1 |
| Producer or Licensed Producer | 1 |
| Producer Registration | 1 |
| Product Lot | 2 |
| Production Site | 1 |
| Program | 7 |
| Program or Hemp Program | 1 |
| Prohibited Varity | 12 |
| Propagate | 1 |
| Propagule(s) | 17 |
| Publicly Marketable Hemp Product | 8 |
| Puerto Rico Department of Agriculture Hemp Program | 1 |
| Puerto Rico Hemp Licensing and Inspection Office | 1 |
| QR code | 1 |
| Qualified Agricultural Producer | 1 |
| Raw Hemp | 2 |
| Reasonable Efforts | 2 |
| Refined Hemp Oil | 1 |
| Registered Land Area | 8 |
| Registered Producer | 1 |
| Registrant | 3 |
| Registration | 2 |
| Remediation | 1 |
| Render Cannabis Non-Retrievable | 1 |
| Representative Sample | 1 |
| Research | 1 |
| Research Area | 1 |
| Research License | 1 |
| Reservation | 1 |
| Reverse Distributor | 10 |
| Sample | 6 |
| Sample Collection Date | 1 |
| Sampler | 1 |
| Sampling | 2 |
| Sampling Agent | 2 |
| Secondary Pre-Harvest Sample | 1 |
| Seed | 2 |
| Seed Distributor License | 1 |
| Seed Source | 5 |
| Sell/Sale | 2 |
| Site | 2 |
| Smoking | 1 |
| Special Hemp Seed Importation Permit | 1 |
| Specimen | 2 |
| State Plan | 1 |
| Sterilization | 1 |
| Storage | 2 |
| Storage Area | 3 |
| Store | 4 |
| Strain | 1 |
| Subcontractor | 3 |
| Temporary Harvest and Transportation Permit | 1 |
| Test or Testing | 1 |
| Test Sample | 1 |
| Testing Facility | 2 |
| Testing Laboratory/Laboratory | 1 |
| Testing THC Hemp | 1 |
| THC | 29 |
| THC and THCa | 1 |
| THC Concentration | 1 |
| THC Free Distillate | 1 |
| THC-A | 3 |
| THCA | 3 |
| Total Delta-9-Tetrahydrocannabinol Concentration | 1 |
| Total Delta-9-THC | 1 |
| Total THC | 3 |
| Transplant | 3 |
| Transport | 1 |
| Transport Manifest | 2 |
| Transporter | 1 |
| Tribal Hemp License | 1 |
| Tribal Hemp Officer | 1 |
| Tribal Hemp Regulation | 1 |
| Unprocessed Hemp Plant Material | 1 |
| Variety | 32 |
| Variety of Concern | 4 |
| Variety or Strain | 3 |
| Viable Industrial Hemp | 1 |
| Viable Seed | 1 |
| Volunteer Cannabis Plant | 10 |
| Volunteer Industrial Hemp Plant | 1 |
| Volunteer Plant(s) | 6 |
| Waste | 1 |
| Waste Disposal Plan | 1 |
| Wild Cannabis | 1 |

A comprehensive table of all terms included in the analysis and the number of plans each term appeared in.
